# Long-term monitoring of GMOs in EU food and feed: a multi-national survey to optimize enforcement strategies

**DOI:** 10.1080/21645698.2026.2668239

**Published:** 2026-05-07

**Authors:** W. Broothaerts, E. de Andrade, R. Hochegger, U. Marchesi, C. Weidner

**Affiliations:** a European Commission, Joint Research Centre (JRC), Geel, Belgium; b National Institute of Agricultural and Veterinary Research (INIAV), Oeiras, Portugal; c Austrian Agency for Health and Food Safety (AGES), Vienna, Austria; d Istituto Zooprofilattico Sperimentale del Lazio e della Toscana M. Aleandri (IZSLT), Rome, Italy; e Federal Office of Consumer Protection and Food Safety (BVL), Berlin, Germany

**Keywords:** Control laboratories, EU regulation, feed, food, frequency, genetically modified organisms, GM events, monitoring, survey, trends

## Abstract

To ensure food safety, consumer protection and environmental sustainability, the European Union enforces a robust regulatory framework for GMO monitoring. This study presents a multi-annual survey to evaluate the effectiveness of EU enforcement strategies in detecting and managing GMO presence across the food and feed supply chain. The study aggregates for the first time data across all EU Member States and product categories (food, feed, seed) over three time periods, providing a holistic view of GMO dynamics. The study investigated the number and type of products subjected to analytical tests but also reveals trends in GMO occurrence over time. These observations underscore the importance of updating detection strategies in control laboratories to address the evolving GMO landscape. By leveraging the study’s findings, EU competent authorities and official control laboratories may tailor their monitoring plans to reflect actual market trends and enhance the efficiency of GMO analysis.

## Introduction

The EU regulations on genetically modified organisms (GMOs) within the food and feed supply chains aim at ensuring food safety, consumer choice, environmental sustainability, and a well-functioning internal market.^1^ As GMOs continue to play a significant role in global agriculture, and to satisfy the demand for traceability, the EU has maintained a regulatory framework to monitor GMO presence in food and feed products as well as in seed lots. Regulation (EU) No 2017/625 on official controls,^2^ which came into force in December 2019, further reinforced the EU’s commitment to comprehensive monitoring of the EU food and feed market. This contributes to compliance with food and feed law, including specific requirements for GMO testing. The regulation requires systematic monitoring of food and feed to detect GMOs, ensure their authorization, and, when applicable, verify labeling according to EU requirements. Its objectives include harmonizing the approach across Member States, providing consumers with correct information on the presence of GMOs, maintaining food and feed safety, while ensuring that any unauthorized GMOs are identified and removed from the market.

The effective implementation of the EU GMO legislation is central to the role of the EU Reference Laboratory for Genetically Modified Food and Feed (EURL GMFF) and the National Reference Laboratories (NRLs). The EURL GMFF plays a pivotal role in providing scientific and technical support to NRLs. It ensures harmonized, validated, and reliable GMO detection methods across the EU.^3–6,24^ In addition, it is also responsible for the organization of proficiency testing and training for NRLs, thus ensuring that laboratories have the necessary skills and tools to carry out their legal tasks effectively.^7^ In this complex scenario, NRLs serve as the interface between the EU and national levels, facilitating continuous horizontal technical cooperation among Member States’ reference laboratories and acting within the vertical transmission chain between the EURL and the national official control laboratories (OCLs).

The OCLs in the EU, supported by the EURL GMFF and the respective NRLs, have an essential function in this regulatory framework by conducting routine analyses to detect and quantify GMOs in food, feed and seed samples. The control data generated by these laboratories provide invaluable insights into the occurrence of GMOs within various sample categories across the EU market. Furthermore, they indicate whether the GMO content in food or feed products complies with the prevailing EU thresholds for the labeling of such products. The continuously increasing number of GMOs on the market, including those that escape common screening targets, puts pressure on the limited resources of GMO analysis laboratories.

This work presents a descriptive evaluation of GMO analysis data collected from control laboratories across the EU during three time periods. While other studies (e.g., Waiblinger et al., 2018, 2023)^8,9^ focused on food samples from the German market, this study uniquely aggregates data across all EU Member States and product categories (food, feed, seed). Using comprehensive data collected at different time periods, trends over time in the type and frequency of certain GMOs are revealed. Such studies are essential for understanding the dynamics of GMO presence in food and feed, assessing the effectiveness of regulatory interventions and instructing risk-based monitoring programs. The outcome may contribute to ensure that the regulatory framework effectively informs and protects consumers and may guide future policy decisions.

## Materials and Methods

### Design and Scope of the Surveys

Three surveys were organized by the EURL GMFF to collect data on official GMO controls performed by OCLs in the EU:

- In 2018, a survey was sent to NRLs and OCLs involved in GMO controls in the EU and EFTA countries Norway and Switzerland. The survey focussed on GM events detected in feed products during 2016–2017. The answering options included “never,” “occasionally” or “regularly.” The survey included an open question on GM events detected in food products, but only a few events, also found in feed, were reported (without information on their frequency) and these data were not included in this study. A total of 55 participants (28 NRLs, 27 OCLs) from 26 EU Member States reported data.
- In 2021, a similar survey was sent to NRLs and OCLs in all EU Member States to collect data on routine samples across product categories covering the period 2019–2020. The question asked was: “*How often were the following GM events identified in routine samples (labelled as GMO or not) in your lab in the past two years?*.” Answering options were “never,” “few times” (=occasionally), “regularly” (clarified as “in +/- 10% of samples”), and “often” (“in >20% of samples”). A total of 60 participants (28 NRLs, 32 OCLs) from 22 EU Member States provided information.
- In 2023, another survey was sent to all EU NRLs with the request to collect comprehensive data on official controls during the year 2022. Additionally, 12 OCLs from Germany, one from Italy (for seed testing) and one from Finland reported results. Data on GMOs in food products were reported by one OCL for the whole of Germany and were based on Waiblinger et al. (2023).^9^ Data reported by Bulgaria and Lithuania were for 2023 instead of 2022, which should, however, not significantly affect the study. The same selection options as in 2021 were given. The survey also collected data on the number of official control samples analyzed in each member state per product category (food, feed, etc.). A total of 51 participants (36 NRLs, 15 OCLs) from 25 Member States reported data, and the majority of respondents confirmed that the data were complete and accurate rather than estimations.

## Statistical Analysis

To enable comparison across surveys, qualitative frequencies were converted into percentages, with a relative weight applied to the categories in all three surveys. To rank GM events by prevalence, a numerical weight was assigned to qualitative responses: “often” was equaled to 20%, “regularly” to 10% and “few times” to 3%, then the weighted data were summed up and ranked from the highest to lowest incidence. For the 2018 survey, which lacked the “often” option, “regularly” was weighed at 15% to reflect its broader interpretation as it may refer to both “often” and “regularly” in the other surveys. Relative weighing factors were calculated by dividing the assigned weighing factor by the number of positive detections of the GM event per survey.

Authorization dates were retrieved from the Biosafety Clearing House (https://bch.cbd.int/en/)^10^ and from the International Service for the Acquisition of Agri-biotech Applications, Inc. (https://www.isaaa.org/).^11^ EU authorization dates were verified using the Food and Feed Information Portal Database of the European Commission.^12^

Statistical trend analysis was performed by use of the software Prism (Version 8.4.3, GraphPad, CA, USA). The relative weighted frequency of GM events and the duration of authorization was tested for correlation with the nonparametric Spearman model (two-tailed, 95% confidence).

## Results

### Official Control Analysis for the Presence of GMOs in the EU

The legal framework to carry out official controls in the EU is Regulation (EU) 2017/625.^2^ These controls include taking samples and testing to verify compliance with marketing authorization and labeling requirements. Labeling of products containing (authorized) GMOs is mandatory in the EU above a threshold of 0.9% (mass of the GM ingredient per total mass of the ingredient/species). A zero tolerance policy applies to GMOs that have not been authorized in the EU. However, a lower technical threshold (0.1%) is applicable in case of the adventitious and technically unavoidable presence of GMOs in feed products when the authorization is pending or has expired^13^ (certain conditions apply). The number of physical controls and laboratory tests performed each year is the responsibility of the individual Member States, while this responsibility is decentralized to the federal states in some federal Member States. The annual planning of the official controls for a variety of substances is dependent on the perceived risks for noncompliance or unsafety to human, animal or plant health, animal welfare or, with regard to GMOs, also to the environment.

The determination of the number of risk-based GMO inspections with laboratory checks in the Member States depends on several factors. The different GMO policies across EU Member States influence the importance attributed to the presence of GMOs in the food and feed chain and thus the number of laboratory controls requested by the authorities. Laboratory controls usually include a larger number of samples and GM events tested when GMOs are considered to pose a higher risk. For instance, the increased risk of noncompliance with GMO regulations for animal feed compared to food may justify testing more feed samples.^14^ On the other hand, from a consumer’s perspective, the presence of GMOs in food products is considered more relevant and perceived more critical than in animal feed. In addition, the number of controls on seeds is particularly higher in countries with extensive organic or GMO-free production, such as Austria, Hungary and Italy, as well as with GM plant cultivation under co-existence conditions (when GM and conventional crops are grown in the same region), such as Spain and Portugal. Consequently, the extent of testing for regulatory purposes exhibits significant variation between Member States.

In 2023, a survey among Member States’ reference laboratories provided a comprehensive overview of official GMO control testing in the EU (Table 1). The data, covering the year 2022, were reported by all 24 EU Member States that have control laboratories within their country. The three missing EU Member States may send their samples to laboratories in other Member States, but this concerns a very limited number of samples per year. It is also noteworthy, that not all Member States analyzed all categories of products. For instance, in 2022, one member state did not test any food products as the focus of the competent authority was on feed products in that particular year. In addition, two Member States did not report any feed testing. A smaller number of Member States performed controls for the other product categories, like seeds, plants and food or feed additives.

**Table 1.** Number of products tested and results of GMO findings across the EU in 2022.

Product category		Food	Feed	Seed	Plants	Feed additives	Food additives	Total
Number of countries		23	22	15	11	10	5	
Total number of products		6624	3321	5235	383	65	101	15729
Negative for GMOs	Number	6280	2048	5165	342	54	92	13981
	%	**94.8**	**61.7**	**98.7**	**89.3**	**83.1**	**91.1**	**88.9**
GMO content ≤ 0.9%	Number	288	807	50^a^	10^a^	4^a^	6^a^	1165
	%	**4.4**	**24.3**	**1.0**	**2.6**	**6.2**	**5.9**	**7.4**
GMO content > 0.9% (to be labelled)	Number	40	421	1^a^	11^a^	2^a^	0^a^	475
	%	**0.6**	**12.7**	**0.02**	**2.9**	**3.1**	**0**	**3.0**
“Pending GMO” content ≤ 0.1% ^b^	Number	NA	10	NA	NA	NA	NA	10
	%	–	**0.3**	–	–	–	–	**0.1**
“Pending GMO” content > 0.1% ^b^	Number	NA	0	NA	NA	NA	NA	0
	%	–	**0**	–	–	–	–	**0**
Unauthorised GMOs	Number	16	35	19	20	5	3	98
	%	**0.2**	**1.1**	**0.4**	**5.2**	**7.7**	**3.0**	**0.6**

NA, not applicable.

^a^Labelling threshold of 0.9 (m/m) % is only applicable to food and feed, not to seeds, plants and food or feed additives produced with a GMO.

^b^GMOs with pending or expired EU authorisation may fall under Regulation (EU) No 619/2011, which stipulates a technical threshold of 0.1 m/m % for feed products.

A total of over 15,700 control samples were tested for the presence of GMOs in 2022. Of these, 6,725 samples were from food products and food additives, 3,386 from feed and feed additives, and 5,618 were from seeds and plant materials (Table 1). Most of the analytical tests (89%) were negative for the presence of GMOs across all sample categories, with the highest negative rate being recorded for seed (98.7%) and food (94.8%). The lowest negative rate was observed for feed (65.6%), which also revealed the highest rate (12.7%) of GMOs above the labeling threshold (>0.9 (m/m) %). Many of these feed samples were correctly labeled as containing GMOs and were thus compliant with the regulatory requirements (see further below). In the context of food products, only 0.6% of the samples were classified above the GMO labeling threshold.

Furthermore, only 10 feed samples contained GM events with pending or expired authorization status according to Regulation (EU) No 619/2011,^13^ and in all these cases, the GMO content was below the technical limit of 0.1 m/m % for adventitious GMO presence. Labeling rules do not apply to seeds, plants, or food/feed additives (*e.g.*, enzymes produced by a GM microorganism), hence the quantitative data reported for such samples are not relevant. In seeds, zero tolerance applies and the data confirm that the large majority (98.7%) of seed samples are found compliant. The plant category comprises a variety of plant species, including maize leaves and maize kernels collected in different areas of Spain and Portugal, respectively, to verify coexistence rules between MON810 (the only GMO approved for cultivation in the EU) and non-GM maize cultivation. All GM-positive plants were detected in these countries. In food or feed additives, the non-compliant findings generally relate to the detection of DNA from the microbial production strains in the final products.

Finally, a total of 98 samples, encompassing all product categories but mainly in feed, were reported as containing GMOs that had not been authorized in the EU. It is important to note that unauthorized events are those that are not listed as authorized in the European Commission’s register at the time of reporting (previously referred to as the GMO register, now the Food and Feed Information Portal Database (FIP).^12^

### Non-Compliant Products on the EU Market

The 2023 survey included questions for reporting cases of products that were found to be non-compliant with the GMO legislation in the EU. Two categories of non-compliances were considered: 1) unlabeled products containing GM events that are authorized in the EU at a level exceeding the labeling threshold (Table 2), and 2) products containing GM events that are not (or not yet or not anymore) authorized in the EU for a given use (Table 3). The 74 reported infringements against the labeling requirements (category 1) were mainly found in various feed products (81%) rather than in food (19%). Of the food products, the majority were imported from outside the EU, whilst within the unlabeled feed category, also several products that were produced in the EU were found. Across all products a variety of GM events were identified, with GM contents ranging from a few percentages to 100 m/m %, predominantly involving soybean events (a more detailed table is available as Supplementary Table S1). The higher occurrence of GM events in soybean compared to maize, oilseed rape or other crops is probably due to the widespread cultivation of GM soybean in the relevant exporting countries and the substantial volumes of soybean imports for satisfying protein needs in EU feed. Infringements of the labeling regulation do not *per se* pose a safety risk as the GM events have been authorized for use in food or feed and are thus considered safe. However, they provide misleading information to consumers, and this should be avoided.

**Table 2.** Reported food and feed products non-compliant with the labelling requirements in the EU (data from 2022).

Category	Number of products	Type of product	Origin of product	GM events detected in order of frequency^a^
Food	14	Processed soybean, soybean lecithin, maize popcorn, corn starch, white and parboiled long grain rice, or undefined	South-Korea, China, Romania, Moldova, Burma, Russia or unknown	40–3-2, MON89788, MON87701, A5547, MON87708, MON810, NK603, 44,406, FG72, MON89034, 1507, MIR162, GA21, MON88017, A2704, Bt11, MON87751, MON87427, MIR604, 305423, 59122, CV127, MON87460
Feed	60	Compound feed for rabbits/reptiles/horses/chicken/laying hen/pig/cattle/ruminants, complementary feed for birds, (cracked) corn for feed, soybean meal, pet feed, or undefined	Germany, Denmark, Croatia, Colombia, Italy, Ireland, USA or unknown	

^a^Data shown relate to all product categories, since only fragmented data were available for each product (more details in Supplementary Table).

**Table 3.** Reported incidences of non-authorized GMOs detected in EU market products (data from 2022).

Category	Number of products	Type of product	Origin of product	GM events or elements detected
Food	2	Rice	Italy, China	p35S, tNOS, p35S:bar; not reported
Food	2	Papaya	Thailand, Cambodia	p35S, tNOS; GM papaya (‘Thai papaya’)
Food	Not reported	Flax (linseed)	Not reported	GM flax FP967
Food enzymes	1	Food enzyme preparation	USA	Construct-specific markers for GMM producing amylase
Feed enzymes	1	Feed additive containing phytase	Unknown	Construct-specific markers for GMM producing amylase & protease
Plants	20	Roadside rapeseed plants	France	GT73, Ms8, MON88302, Rf3

The survey revealed detailed information for only a few products containing unauthorized GMOs (Table 3), including rice and papaya positive for genetic screening markers, and one food and one feed additive composed of enzymes produced by GM microorganisms (GMM) being positive for a construct-specific element.^15,16^ In addition, GM flax was also mentioned by two laboratories as being detected a “few times,” but no further details were provided. Furthermore, a French study identified several rapeseed plants along roadsides that contained one of four different GM rapeseed events for which no authorization for release in the environment exists. The comparatively low number of specific cases of products containing unauthorized GMOs for which details were reported in the survey contrasts with the much higher numbers reported in Table 1. It may be assumed that the participants of the survey had easily access to the total number of infringement cases, but that providing details of these cases would have required a more time-consuming investigative process. In case a potential violation is not considered to present a risk, it may be notified in the Administrative Assistance and Cooperation Network (AAC; https://food.ec.europa.eu/food-safety/aac_en), allowing to share the information with other Member States. For instance, Germany reported almost half of the findings of unauthorized GMOs (47) but only provided details concerning a single papaya product. In the notifications under the rapid alert system for food and feed of the EU ^17^ only seven products were reported in 2022, including GM papaya, rice noodles, linseed and two food/feed additives. A more elaborated review of the RASFF notifications on GM food and feed between 2002 and 2023 can be found in Eissa et al. (2024).^18^

### Type and Frequency of GM Events Identified in Control Samples

The surveys conducted among official control laboratories in the EU Member States also provided details about the GM events detected across all product categories. For each GM event, authorized in the EU or with pending or expired authorization status at the time of the survey, the laboratories were requested to report the frequency of the positive detections. The qualitative options to select were “never,” “few times,” “regularly,” *i.e*., in approximately 10% of samples, or “often,” *i.e*., in more than 20% of samples (the latter option was not included in the 2018 survey). While a majority of laboratories provided an indication on the occurrence for every GM event, a few laboratories only indicated those events that were detected at least a few times, while never detected events were left unanswered. Consequently, a proportion of the “no answer” results may in fact refer to “never” detected answers, although in most cases the empty answer indicates that the presence of the GM event was not tested in the laboratory, *e.g*., because the detection method for that GM event had not (yet) been implemented.

The most recent survey, covering the year 2022, included 26 maize events, 17 soybean events, 9 oilseed rape events, 14 cotton events, and 1 sugar beet event. In addition, data were requested on (non-authorized) GM rice, GM papaya and GM flax. Two of the maize GM events had an expired authorization status in the EU (Bt176 and MON863), and for one event (98140 maize) the EU authorization dossier had been retracted by the applicant, although a validated detection method and CRM are available. Fifty-six laboratories in 24 EU Member States provided data on GMO occurrences. These data were collected and reported by the NRL(s) in the Member State on behalf of all control laboratories or they were contributed by the individual control laboratories themselves. *E.g*. Germany had 14 contributions, but all data on food were reported by one laboratory, while Belgium, France, Poland and Spain, each had three contributions, and Bulgaria, Italy and the Slovak Republic each had two. The remaining Member States were represented by data reported by a single laboratory. The laboratories performed control analyses on a variety of products, with some conducting analyses on all types of products, while others specialized in food, feed or seed samples.

Genetically modified soybean, maize, and oilseed rape events were found by several laboratories in different countries, with varying frequencies (Table 4). Conversely, because GM cotton and GM sugar beet were never detected by any laboratory (data not shown), only about half of the laboratories routinely tested for the presence of these GM crops in market products. Among the widespread crops with GM varieties (*i.e.*, maize, soybean and oilseed rape), 12 out of 52 GM events (23%) were “never” detected by any respondent laboratory. These include the maize events MZHG0JG, MZIR098, MON87429, 3272, MON95379 and 98140; the soybean events GMB151 and SYHT0H2; and the oilseed rape events T45, MS11, MON94100 and LBFLFK. It is noteworthy that the majority of these events (except for 98140, which has been retracted, and 3272, MS11 and LBFLFK, for which the authorization is pending) have been authorized relatively recently in the EU (in 2019 or later). The frequency of the remaining events varied between countries and laboratories, *i.e.*, the same event could be detected “few times,” “regularly” or “often,” although the more widespread events were often detected by many laboratories and the rare events by only a few.

**Table 4.** Frequency of GM events (soybean, maize, and oilseed rape) detected in EU market products expressed by number of laboratories (data for 2022).

Crop	GM event	Never	Few times	Regularly (≈10%)	Often (>20%)	No Answer	Total
Soybean	**40–3-2**	5	13	11	18	9	56
	**MON89788**	5	13	14	15	9	56
	**MON87701**	11	12	8	14	11	56
	**MON87708**	14	15	10	4	13	56
	**A5547**	14	22	6	2	12	56
	**44406**	11	25	8	1	11	56
	**A2704**	17	24	3	0	12	56
	**MON87751**	25	11	3	0	17	56
	**FG72**	23	19	2	0	12	56
	**81419**	29	13	1	0	13	56
	**MON87705**	35	7	1	0	13	56
	**305423**	36	6	0	0	14	56
	**68416**	40	3	0	0	13	56
	**CV127**	39	3	0	0	14	56
	**MON87769**	37	2	0	0	17	56
	**GMB151**	40	0	0	0	16	56
	**SYHT0H2**	37	0	0	0	19	56
Maize	**MON810**	23	19	5	0	9	56
	**NK603**	24	20	4	0	8	56
	**1507**	21	19	4	0	12	56
	**MON89034**	26	17	4	0	9	56
	**MON88017**	30	11	4	0	11	56
	**MIR162**	29	10	3	0	14	56
	**GA21**	24	19	2	0	11	56
	**Bt11**	26	16	1	0	13	56
	**4114**	34	6	1	0	15	56
	**5307**	40	3	1	0	12	56
	**MIR604**	31	13	0	0	12	56
	**59122**	33	12	0	0	11	56
	**T25**	33	9	0	0	14	56
	**MON87427**	32	8	0	0	16	56
	**MON87411**	32	7	0	0	17	56
	**40278**	39	5	0	0	12	56
	**MON87460**	35	5	0	0	16	56
	**MON87403**	37	2	0	0	17	56
	**MZHG0JG**	36	0	0	0	20	56
	**MZIR098**	36	0	0	0	20	56
	**MON87429**	37	0	0	0	19	56
	***3272***^***a***^	40	0	0	0	16	56
	**MON95379**	34	0	0	0	22	56
	***98140***^***b***^	41	0	0	0	15	56
	***Bt176***^***b***^	41	2	0	0	13	56
	***MON863***^***b***^	40	5	0	0	11	56
Oilseed rape	**GT73**	18	19	3	3	13	56
	**MON88302**	26	12	0	1	17	56
	**Rf3**	30	10	0	1	15	56
	**MS8**	31	9	1	0	15	56
	**73496**	39	1	0	0	16	56
	**T45**	40	0	0	0	16	56
	***MS11***^***a***^	37	0	0	0	19	56
	**MON94100**	33	0	0	0	23	56
	***LBFLFK***^***a***^	31	0	0	0	25	56

^a^Authorisation is pending, falling under Regulation (EU) No 619/2011.

^b^Authorization has expired.

**Figure 1.**
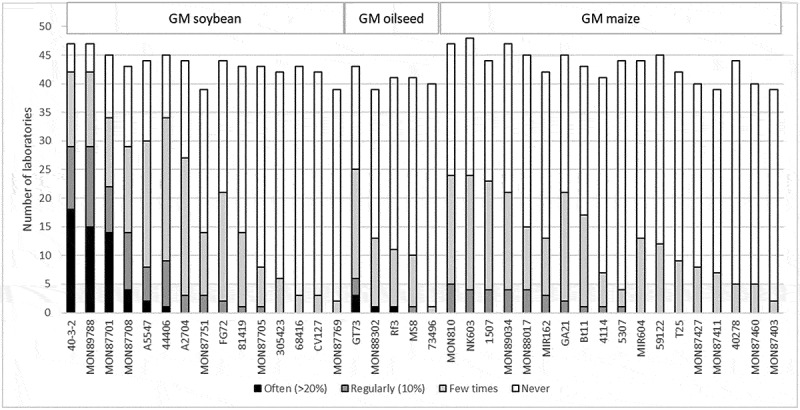
Frequencies of GM events detected by official control laboratories on the EU market in 2022, the most prevalent GM event per crop is 40–3-2 soybean, GT73 oilseed rape, and MON810 maize.

Figure 1 shows the reported frequencies of GM events across all crops for those events that were at least a “few times” detected by the control laboratories in 2022 (excluding the “no answer” category). Among the events that were detected “often,” only GM soybean and GM oilseed rape were found, while none of the 26 maize GM events were detected more than “regularly.” Notably, GM soybean 40–3-2 and oilseed rape GT73, both first authorized for food, feed and seed in the USA before the year 2000, and in the EU in 2005 and 1997, respectively, had the highest prevalence within their respective plant species, with 42 and 25 laboratories reporting their detection, respectively. In soybean, the events 40–3-2 and MON89788 were reported by the same number of laboratories, but 40–3-2 was identified by 18 laboratories as present in more than 20% of the samples (“often”), whereas MON89788 was reported by only 15 laboratories in the same frequency. Although the GT73 event was identified as the most prevalent among oilseed rape events, it was only detected “often” by three laboratories.

For GM maize, only a few laboratories detected several events regularly, *i.e*., with a frequency of approximately 10% of the samples. The highest frequencies were recorded for MON810, NK603, 1507, MON89034, and MON88017, in descending order. These events were, however, detected by less than half of the laboratories, while the other half never detected them. The MON810 event has been authorized in the EU for over 25 years, NK603 and 1507 since 2004 and 2005, respectively, and the other two events since 2009. It therefore appears that the oldest GM maize events are still the most abundant ones on the EU food and feed market.

The correlation between the year of authorization, either in the EU or outside, and the occurrence of GM events on the EU market was further investigated. From a global perspective, 37 events have been authorized for over a decade for food or feed use or cultivation in at least one country (often first in the US), with 15 of these events exceeding two decades. In the EU, 15 events have been authorized for more than 10 years (for food or feed use, and only MON810 also for cultivation), with only 5 exceeding two decades.

**Figure 2.**
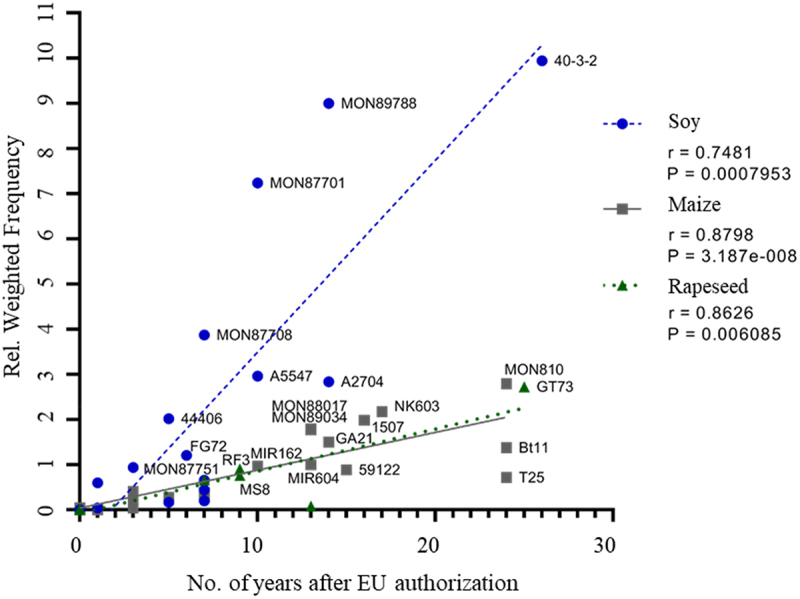
Correlation between EU authorisation duration and relative weighted frequency of GM events for soybean (blue circle, dashed line), maize (grey square, solid line) and rapeseed (green triangle, dotted line) on the EU market in 2022. Expired GM events were omitted from the analysis. Linear regression line and Spearman correlation data are shown for the three species separately (soybean *n* = 17; maize *n* = 23; rapeseed *n* = 9). r, correlation coefficient; P, *p* value for Spearman correlation analysis (95% confidence, two-tailed).

To compare the reported answers on GM event detections, a numerical weighting factor was attributed to each of the answers, then summed up per event and expressed in relation to the total number of positive answers for that event (see Materials and Methods). The observed variation in the relative weighted frequency between events was then compared to the number of years after the first authorization in a third country or in the EU. There was a clear correlation between the detection of the GM events and the period of authorization in the EU (related to reporting year 2022) (Figure 2). The correlation was statistically significant for all species, with maize revealing the clearest trend. The correlation was less significant when the occurrence of GM events on the EU market was compared to the year of the *first* authorization (*i.e*., outside the EU) (Figure S1). The three most prevalent maize events are all authorized in the EU for more than 17 years, while for soybean the most prevalent event (40–3-2) is authorized since 26 years, and the two other prevalent events since 14 years (MON89788) and 10 years (MON87701). Also, GT73 oilseed rape is authorized for 25 years. This confirms that the EU food and feed market is still dominated by the older GM events.

### Long-Term Trends in GMO Occurrences

The occurrence of GM events within the EU market depends on many factors, including the year of authorization, worldwide cultivation area and volumes of import into the EU, which may change over the years. The EU itself is not growing a significant volume of GMOs, except almost 70,000 hectares of MON810 maize in Spain and Portugal.^19^ To highlight and interpret possible changes and trends, the GMO frequencies reported for 2022 were compared with similar data obtained from two previous surveys covering 2016–17 and 2019–20. It should be noted that the earlier surveys were not as comprehensive as the 2022 survey, in the sense that they did not cover all control laboratories in all EU Member States. However, the relative frequencies of GMO detections are considered comparable across years.

**Figure 3.**
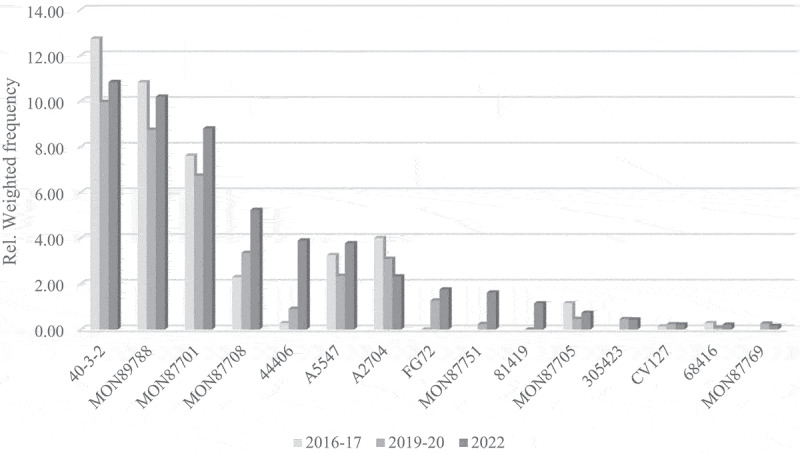
Long-term trends in GM soybean occurrence on the EU market.

**Figure 4.**
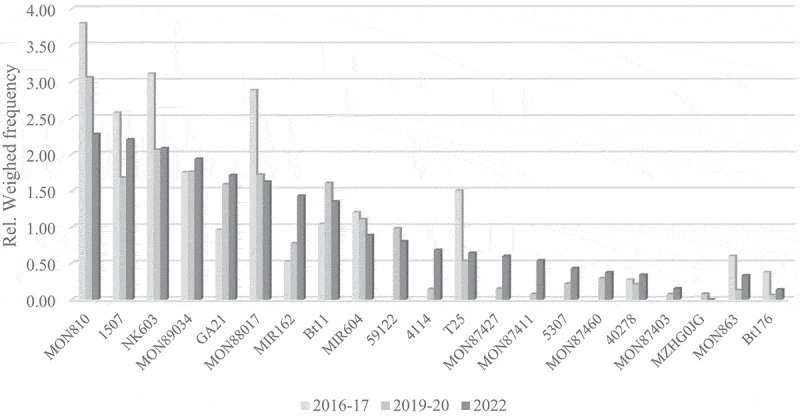
Long-term trends in GM maize occurrence on the EU market.

**Figure 5.**
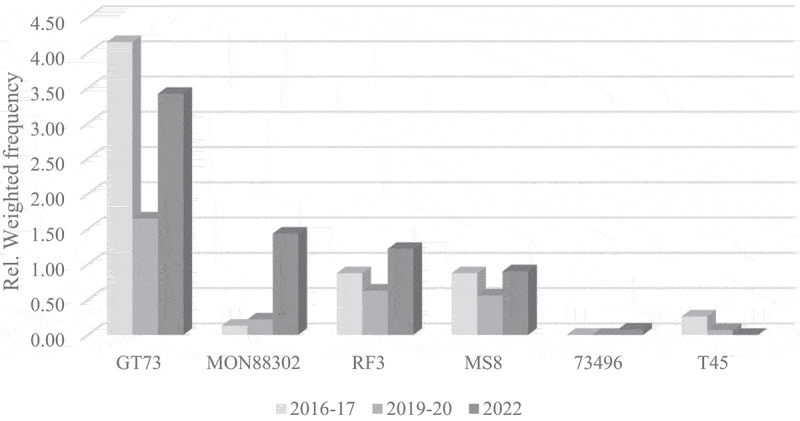
Long-term trends in GM oilseed rape occurrence on the EU market.

A general observation reveals that, for all crop species subject to official control in the EU, the ranking of the most frequently detected GM events authorized in the EU remains rather stable over this short period of seven years (2016–2022). This finding is substantiated through a comparative analysis of the relative weighted frequency of GM events across the three crop species over three temporal periods (Figures 3–5). The GM events are shown in order of importance on the EU food and feed market in 2022, omitting events that were never detected so far. For certain recently authorized or minor GM events no data are available for 2016–2017.

With regard to GM soybean, the top three events 40–3-2, MON89788, and MON87701 reveal a consolidated high ranking across time. Furthermore, a strong increase in the occurrence of MON87708 and 44406 is observed, while A2704 gradually lost market share in the same period. Among the minor events, the emergence of FG72, MON87751 and 81419 is of note.

For GM maize, the events MON810, NK603 and 1507 are consistently found in the top three places, although the frequent detection of MON810, in particular, has dropped to the same level as the other two events in recent years. MON89034 remained steady on the fourth place over the observed time period, while MON88017 has dropped to the same level after a high abundance in the first survey. Conversely, GA21 and MIR162 have shown an increase in frequency in recent years. Furthermore, the onset of new emerging maize events such as 4114, MON87411 and 5307 is noteworthy. Finally, the expired maize GM events MON863 and Bt176 have seen an expected further decline in share in the EU in recent years.

Similarly, for oilseed rape, the GT73 event keeps the first position, although with a very peculiar trend over the years. A comparison of the 2016 survey with the two most recent surveys demonstrates a reduction in the number of laboratories detecting this event (from 77% in 2016 to 44% in 2019 and 58% in 2022), but at the same time the event is detected more frequently in these laboratories. MON88302 strongly increased in detection frequency in the last survey and moved into second place, replacing the hybrid seed production players RF3 and MS8.

## Discussion

The analysis of GMO occurrence in food and feed samples across the EU, based on data collected in 2018, 2021, and 2023, reveals significant trends that have important implications for regulatory monitoring and enforcement. The data indicate that while older GMOs continue to be prominently detected, mostly in feed samples, there is a noticeable tendency for the emergence of newer GMOs on the market. The most recent evidence indicates that the detection of newly introduced GMOs in commercial products begins to occur around a decade after their authorization and entry into the global market, suggesting a lag between market introduction and widespread detection in the EU. This 10-year lag in occurrence on the EU market is believed to be caused by regulatory approaches of biotech companies seeking authorization in North-American markets first but delaying cultivation until authorization is obtained in South-America and in export countries, like the EU. The EU authorization process also generally takes longer than in many other countries and the need for validated detection methods adds up to this timeline. Interestingly, the most important GM soybean events identified on the EU market in this study (40–3-2, MON89788 and MON87701) somewhat differ from those reported in other studies. For instance, Soga et al.^20^ reported the prevalence of MON89788 and MON87708 soybean in harvests from the US and Canada collected by Japanese trading companies in 2021 and 2022, and showed these to mainly occur as a GM stack. A5547 and 44406 soybeans were also found in all samples, while 40–3-2 and MON87701 soybean were only detected in some of the samples and at lower levels.^20^ The results may be specific for the trading from North-America to Japan, while the European data analyzed in this study include imports into the EU from a wider variety of source countries. The observed dynamism in GM event occurrences pose challenges to continuously updating surveillance and control approaches. This was previously also concluded by ^9^ on the basis of the German official food control analysis.

In the majority of EU Member States, the analysis of GMOs is typically conducted using a modular approach. This approach involves the preliminary screening for the presence of common genetic elements and constructs, with the objective of minimizing the subsequent number of event-specific analytical tests required.^21^ A range of tools have been developed for the evaluation of the screening results (*e.g.*, JRC GMO-Matrix: https://gmo-crl.jrc.ec.europa.eu/jrcgmomatrix/matrices/,^22^ EUginius GMO analysis tool, BVL screening table: BVL – Startseite – Screeningtabelle für den GVO-Nachweis 2024).^23^ There has been a substantial increase over the years in the number of GM events to be screened for and also in the number of GM events that are negative for all common screening elements. The latter can only be detected with the event-specific methods complementing the first-line screening approach. This poses a significant challenge to the monitoring capacities of the control laboratories.^8,^^9^ Particularly, laboratories testing animal feed are likely to be confronted with a variety of GMOs, including those that have only recently been approved. In contrast, laboratories testing food usually obtain negative results in all analyses. The number and type of screening tests applied by different laboratories vary considerably, and hence also the coverage of GM events in these tests. Some laboratories will, consequently, detect a broader variety of GM events, and also have a higher chance of detecting unauthorized events. In the majority of cases, unauthorized or rare events are usually found at trace levels only, with negligible risks for consumers.

The data collected in the surveys presented here are derived from the official controls carried out in the EU Member States. It should be noted that, although these Member States are subject to a harmonized framework of European legislation, each state interprets its control activity according to the characteristics of its own agri-food system (agricultural vocation, import-export, internal market including consumer sensitivity and its political repercussions). This approach is subject to certain inevitable biases. The number and types of samples may vary significantly from country to country, as well as the species that are more or less intensively monitored or the type of supply chains under special control (organic, GMO-free, value chains in general). The allocation of economic and logistical resources by Member States, and the way in which these are used (e.g., the balance between controls for labeling compliance or for the presence of unauthorized GMOs) depend on all the factors mentioned above and can also vary considerably between Member States. The annual control plans, as set by the competent authorities, may also vary from year to year. It should also be mentioned that the implementation of detection methods for new events also requires a certain time in the laboratory, so that even events that are on the market shortly after their authorization are not necessarily detected across all Member States. Considering that the EU is a single market for trading products, the GMO findings and trends reported here are believed to be valid for the EU as a whole, even while the extent of monitoring varies across Member States.

Furthermore, certain Member States do not test feed samples that are already labeled as containing GMOs or do not perform event-specific tests for all GM events if one of the events is already well above the labeling threshold. Moreover, some laboratories only reported detected GM events while leaving the “never (detected)” category blank, meaning that “no answer” could include some of the “never (detected)” cases. Detailed analysis of the 2023 survey indicated that only four laboratories reported frequencies (“few times”) for a limited number of GM events and left the other cells empty. These laboratories represented low to medium throughput laboratories (<100 or <500 samples), and, hence, the general trends would not be affected. It is also noteworthy that there was a variation in the design of the surveys and their participants across different surveys, and the scoring options differed between the first survey and the subsequent ones. We addressed some of the survey-related shortcomings by calculating relative weighing factors for the GMO findings, turning qualitative scoring into quantitative data and taking into account the number of answers per event. On the other hand, the most recent survey is regarded as comprehensive for the entire EU, as all Member States performing official controls were represented, and the majority of contributing laboratories confirmed the accuracy of the data. Despite the limitations, the conclusions drawn here are considered a good representation of the GMO occurrence on the EU market.

The persistence of older GMOs suggests that routine testing for these events should remain a priority in monitoring plans. Simultaneously, the increasing occurrence of newer GMOs indicates the need for control laboratories to extend their screening protocols to include these emerging GMOs. This dual approach will ensure comprehensive coverage and detection capabilities, allowing for timely identification and management of both known and emerging GMOs within the EU market. In view of the resources necessary for the continual extension of analytical methods covered by a laboratory, monitoring plans should also promote collaboration between control laboratories and allow for a dynamic adaptation of screening approaches, particularly for GM elements, considering country-specific factors as import patterns and commodity flows.

Moreover, the data underscore the need for ongoing research and collaboration at the EU level to stay ahead of the evolving GMO landscape. It is essential for regulatory bodies to regularly review and update their monitoring strategies in response to observed trends in occurrence of GMOs. This may involve revisiting and harmonizing analytical screening sets, enhancing laboratory capacities, and fostering greater cooperation among Member States to share knowledge and resources effectively. The network of NRLs and the European Network of GMO laboratories (ENGL), both of which are managed by the EURL GMFF, serve as invaluable meeting and discussion communities for this purpose. In reality, knowledge of the frequencies at which GM events are detected during official analytical controls, enables the competent authorities to better update the criteria for sampling planning (e.g. increase/decrease the number of samples or introducing crop-specific control plans, based on previously observed GM event prevalence rates and the products country of origin). Furthermore, knowledge of the most frequent GM events in products subject to control helps laboratories to optimize their analytical workflow accordingly, from screening strategies to the analytical tests for identification and quantification in which it is most worthwhile to invest. Looking ahead, monitoring plans could benefit from the use of data-driven or AI-supported approaches by integrating the information concerning GM event frequencies. This would allow the efficient optimization of sampling strategies and laboratory workflows based on real-world relevance.

In conclusion, the data collected over the three survey periods illustrate the dynamic nature of GMO presence in the EU food and feed market. The findings underscore the importance of maintaining robust, adaptive, and forward-looking monitoring systems to ensure food and feed safety within the EU. By leveraging the insights gained from these surveys, EU competent authorities and OCLs can refine their monitoring plans, ensuring they remain responsive to both persistent and emerging GMO risks. The continuously increasing number of GM events to be monitored, including many new events that bear none of the currently applied screening elements, emphasize the importance of a risk-based monitoring to keep the enforcement system efficient and fit-for-purpose under the limited available resources. Continuing the collection of monitoring data in a standardized way be one option to support such a risk-based approach. Through sustained vigilance and collaboration, the EU can fulfill its policy commitment to informing consumers about the presence of GMOs in food and feed, and to identify and prevent GMOs that have not (yet) been authorized to occur on the EU market.

## Supplementary Material

Supplemental Material
