# Supplementary material for: Long-term monitoring of GMOs in EU food and feed: a multi-national survey to optimize enforcement strategies
Source: GM Crops Food. 2026 May 7;17(1):2668239. doi: 10.1080/21645698.2026.2668239 (PMC13170376; doi:10.1080/21645698.2026.2668239)
Supplement: Supplemental Material [file KGMC_A_2668239_SM2188.docx]

**Supplementary information**

Table S1. Detailed overview of reported products non-compliant with the labelling requirements in the EU

| **Type of product** | **Number of products** | **Origin** | **GM events detected** | **GM %** |
| --- | --- | --- | --- | --- |
| Food (4), feed (4), 1 PET food sample | 9 | Various | MON87701, MON89034, TC1507, DAS 59122, Bt11, MON88017, MON89788, NK603, MIR162, MON810, MON 87427, MON/GTS 40-3-2, GA21, MIR604, MON87708, DAS-44406-6, FG72, 4114, MON 87460 | from ~3 % to ~ 65% |
| Compound feed (1), compound feed for rabbits (1), reptiles (1), horses (1) | 4 | Germany, USA or unknown | 1. MON89788 2. GTS40-3-2, MON87701 3. 305423, A2704-12, A5547-127, DAS44406-6, FG72, GTS40-3-2, MON87708, MON89788. 4. MON87701, MON89788 | 1. ~38% 2. ~80%, ~34% 3. 0.6%, 0.8%, 1.6%, 11.9%, 0.85%, 11.47%, 56.09%, 40.14%. 4. 84.9%, 42.2% |
| Food: Soybean meal (2), feed for pig (1) or cattle (1) | 4 | South Korea (food), Germany, Denmark | 40-3-2, MON89788, A2704, A5547 40-3-2, MON89788, MON87701 40-3-2, MON89788 40-3-2, MON89788 | 3.0%, 32%, detected, detected, 73%, 14%, detected, 54%, 39%, 1.8%, 0.3% |
| Processed soybean (2), corn starch (2) | 4 | Soybean: unknown (probably Arabian country), corn starch: Syria | Soybean: MON89788, GTS 40-3-2, MON87708, MON87751, A2704-12, MON87701, BPS CV127, A5547, FG72, DAS 44406-6, maize: TC1507, NK603, MON810, MON88017, MON89034, MON87427, Bt11, MIR162, GA21, DAS-40278-9 | > 10 % (each) |
| Feed | 1 |  | MON89788 and MON87701 | >0.9% |
| Chicken feed (3) | 3 | unknown | MON89788, GTS 40-3-2, MON89788, MON87701, GTS 40-3-2, A 5547-127 | 14.7%, 11.5%, 32.5%, 70.6%, 1.8%, 7.3% |
| Compound feeds (4) | 4 | unknown | GTS40-3-2, MON87701, MON89788, MON87708 in all 4 cases | 3.5, 2.8, 1.4, 3.0 |
| Feed mixture | 1 | Hungary | RR soy 40-3-2 | 5.44% |
| Feed | 9 | Italy | Bt11, DAS1507, DAS59122, GA21, MIR162, MON810, MON87427, MON88017, MON89034, NK603, A5547, DAS44406, MON40-3-2, MON87701, MON87708, MON89788 | between 0.21% and 50.7% |
| Maize grains | 1 | Russia | GTS 40-3-2, MON87701 and MON89788 | GTS 40-3-2 (11.99% ± 0.65), MON87701 (1.89% ± 0.08), MON89788 (0.37% ±0.01) |
| Chips (4), laying hen feed (1), ruminant feed (8) | 13 | USA or unknown | MON89034, NK603, TC1507, MON89788 & MON87701 | 80%, 36%, 91%, 98%, 3.8%, 4.3%, 2.5%, 7%, 3.5%, 1.9%, 53.7%, 18.5%, 1.6% |
| Parboiled long grain rice (1), long grain white rice (1) | 2 | Birma | 1) MON89788, 2) MON89788 | 1) 35.1 +/- 9.8 %, 2) 23.2 +/- 6.5 % |
| Feed/feed material | 1 | Soybean | 40-3-2, MON89788 | > 0.9% |
| Soybean food and feed, maize-popcorn | 6 | Romania, Rep. Moldova or unknown | 40-3-2, MON810 | 10%, 24%, 2%, 15%, 98%, 3% |
| Feed mixture NON-GMO (3), feed mixture not labelled | 4 | Serbia, Slovakia or Czech Republic | MON89788, GTS 40-3-2 | 100%, 49%, 21%, 10% |
| Soy lecithin (1), feed sample (1) | 2 | China, Croatia | Soy lecithin: MON89788, MON87708, A5547, DAS44406, FG72, MON40-3-2, MON87701, MON87751. Feed sample: 40-3-2, MON87701, MON89788. | Soy lecitin: 64%±19% MON89788. Feed sample: 1.53% ± 0.46% MON40-3-2. |
| Complementary feed for birds (2), corn for feed, cracked corn for feed, compound feed | 5 | Colombia | MON810, NK603, BT11, MON810, MON810 | 3.88; >1%; >5%; >5%; 3.44%±0.55% |
| Feed | 1 | Ireland | MIR162, MON87701, MON89788, MON87769, FG72, A5547, DAS44406, 40-3-2, NK603, MON89034, GA21, MON810, MON88017, Bt11 | >7.9 % MIR162 |
| **Total number of products** | **74** |  | | |


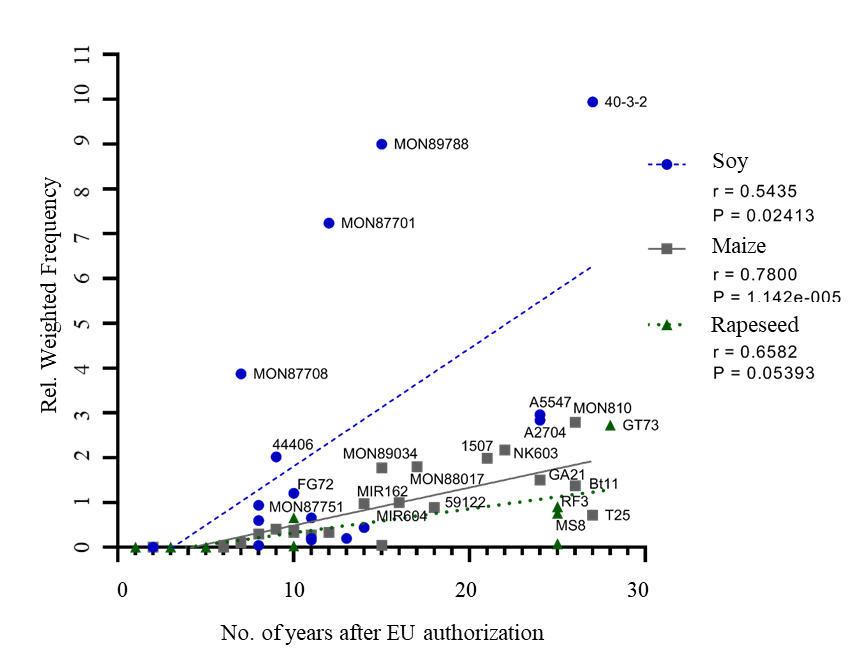


Figure S1. Correlation between worldwide authorisation duration and relative weighted frequency of GM events for soybean (blue circle, dashed line), maize (grey square, solid line) and rapeseed (green triangle, dotted line) on the EU market in 2022. Expired GM events were omitted from the analysis. Linear regression line and Spearman correlation data are shown for the three species separately (soybean n = 17; maize n = 23; rapeseed n = 9). r, correlation coefficient; P, P value for Spearman correlation analysis (95 % confidence, two-tailed).
